# A Primer for Utilizing Deep Learning and Abdominal MRI Imaging Features to Monitor Autosomal Dominant Polycystic Kidney Disease Progression

**DOI:** 10.3390/biomedicines12051133

**Published:** 2024-05-20

**Authors:** Chenglin Zhu, Xinzi He, Jon D. Blumenfeld, Zhongxiu Hu, Hreedi Dev, Usama Sattar, Vahid Bazojoo, Arman Sharbatdaran, Mohit Aspal, Dominick Romano, Kurt Teichman, Hui Yi Ng He, Yin Wang, Andrea Soto Figueroa, Erin Weiss, Anna G. Prince, James M. Chevalier, Daniil Shimonov, Mina C. Moghadam, Mert Sabuncu, Martin R. Prince

**Affiliations:** 1 Department of Radiology, Weill Cornell Medicine, New York, NY 10065, USA; chz4009@med.cornell.edu (C.Z.); xih4004@med.cornell.edu (X.H.); zsh4001@med.cornell.edu (Z.H.); hrd2001@med.cornell.edu (H.D.); uss4002@med.cornell.edu (U.S.); vab4003@med.cornell.edu (V.B.); ars4017@med.cornell.edu (A.S.); moa4017@med.cornell.edu (M.A.); djr4003@med.cornell.edu (D.R.); kut2002@med.cornell.edu (K.T.); hfn4001@med.cornell.edu (H.Y.N.H.); yiw4017@med.cornell.edu (Y.W.); andrea.soto9@upr.edu (A.S.F.); erw4004@med.cornell.edu (E.W.); annaprince2005@gmail.com (A.G.P.); mch4003@med.cornell.edu (M.C.M.);; 2 Meinig School of Biomedical Engineering, Cornell University, Ithaca, NY 14853, USA; 3 Cornell Tech, Cornell University, Ithaca, NY 10044, USA; 4 The Rogosin Institute, New York, NY 10021, USA; jdblume@nyp.org (J.D.B.); jac9014@nyp.org (J.M.C.); das2041@nyp.org (D.S.); 5 Department of Medicine, Weill Cornell Medicine, New York, NY 10065, USA; 6 School of Electrical and Computer Engineering, Cornell University, Ithaca, NY 14853, USA; 7 Vagelos College of Physicians and Surgeons, Columbia University Irving Medical Center, New York, NY 10032, USA

**Keywords:** autosomal dominant polycystic kidney disease, radiological report, semantic segmentation

## Abstract

Abdominal imaging of autosomal dominant polycystic kidney disease (ADPKD) has historically focused on detecting complications such as cyst rupture, cyst infection, obstructing renal calculi, and pyelonephritis; discriminating complex cysts from renal cell carcinoma; and identifying sources of abdominal pain. Many imaging features of ADPKD are incompletely evaluated or not deemed to be clinically significant, and because of this, treatment options are limited. However, total kidney volume (TKV) measurement has become important for assessing the risk of disease progression (i.e., Mayo Imaging Classification) and predicting tolvaptan treatment’s efficacy. Deep learning for segmenting the kidneys has improved these measurements’ speed, accuracy, and reproducibility. Deep learning models can also segment other organs and tissues, extracting additional biomarkers to characterize the extent to which extrarenal manifestations complicate ADPKD. In this concept paper, we demonstrate how deep learning may be applied to measure the TKV and how it can be extended to measure additional features of this disease.

## 1. Introduction

Autosomal dominant polycystic kidney disease (ADPKD) is the most common inherited cause of kidney disease, affecting 12 million globally, with a median age of onset of end-stage kidney disease of approximately 60 years [1]. Timely identification of patients at risk of rapid progression enables early treatment with tolvaptan [2], the only FDA-approved drug treatment that can attenuate the progression of ADPKD.

The total kidney volume (TKV), quantifiable via radiological imaging, is an early indicator of the disease burden [3]. Its significance was underscored initially by the Consortium for Radiologic Imaging Studies of Polycystic Kidney Disease (CRISP) [4]. Consequently, the height-adjusted TKV (htTKV) and its annual rate of change have become primary endpoints in evaluating the treatment efficacy in clinical trials. Recent strides in artificial intelligence (AI) have enabled rapid and reproducible automated TKV measurements [5,6,7,8,9]. However, efforts to integrate TKV evaluation into clinical practice remain limited, as indicated by the PKD Foundation’s 2022 survey, which revealed that 69% of patients had never undergone TKV measurements, and in those who had, the volume was measured predominantly for research purposes [10]. This gap in clinical application persists despite efforts to incorporate TKV quantification into clinical management [6,8].

**Figure 1:**
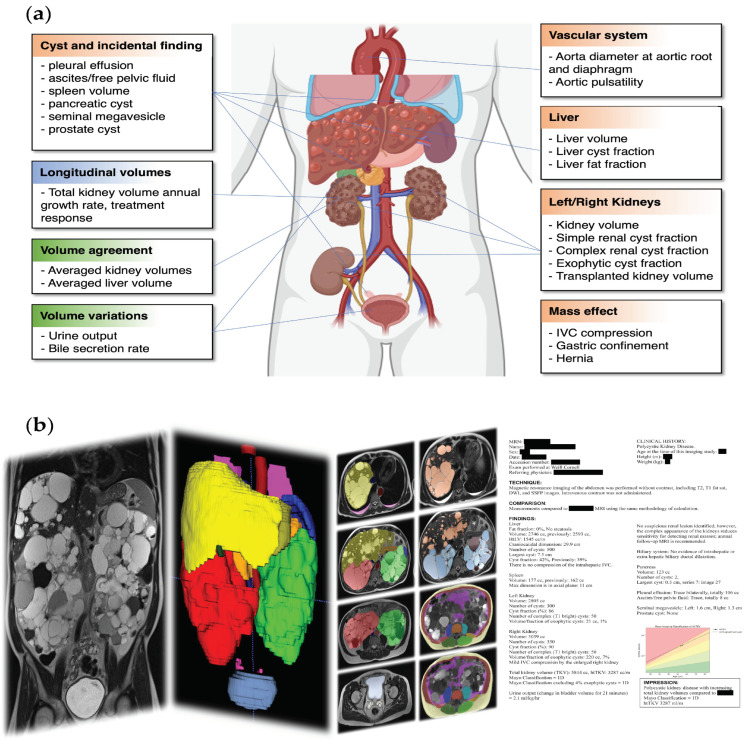
(**a**) Features and complications of ADPKD. Metrics derived from longitudinal MR exams (blue), from multiple image sequences within one MR exam (green), and from a single image sequence (brown) are indicated in boxes; (**b**) image analysis pipeline transforming images (left panel) into segmentation (middle panels) and a radiological analysis report (right panel). From left to right, there is one coronal SSFSE image covering the abdomen to the pelvis, and then the liver (yellow) and kidneys (right = red, left = green) are shown to be enlarged in a 3D rendering of segmentation. The third panel shows the segmentation of multiple anatomical structures on axial images, including organs, tissues (fat and muscles), and cystic pathology. In the fourth panel, there is a radiological report with protected health information redacted.

ADPKD has numerous extrarenal manifestations that contribute valuable prognostic data and aid in disease monitoring [11,12]. These include cardiovascular complications, fluid accumulation, and mass effects due to enlargement of the kidney(s) and liver, all detectable on magnetic resonance imaging (MRI) [13]. Consequently, MRI is well-positioned to contribute to the comprehensive and personalized assessment of ADPKD (Figure 1a). In practice, however, relevant imaging details are often excluded from clinical radiological reports due to the technical challenges of interpreting cystic organs and the time required to manually segment images for TKV calculation. Although radiological imaging of ADPKD has been extensively reviewed [13,14,15,16,17], a workflow that integrates extrarenal quantitative imaging metrics has not been practically implemented.

Herein, we propose a workflow for extracting quantitative data from abdominal–pelvic MR images facilitated by a deep learning organ segmentation model (Figure 1b). We delineate the process, beginning with image acquisition, followed by automated segmentation of organs or tissues, and concluding with the formulation of a detailed radiological report. We currently employ this approach, which provides an effective strategy for ADPKD management that we believe is generalizable to the ADPKD patient population.

## 2. Image Acquisition

### 2.1. MRI vs. Ultrasound and CT for Longitudinal ADPKD Assessment

MRI has advantages over ultrasound due to its more extensive field of view—essential for measuring enlarged cystic kidneys and other affected organs—coupled with a superior spatial resolution, enhanced image contrast, and lower operator dependence. Although computed tomography (CT) shares the benefits of a broad field of view and high resolution, it requires ionizing radiation at doses that can become considerable cumulatively over a lifetime of periodic imaging [18]. Moreover, CT requires intravenous infusion of iodinated contrast agents to optimize image contrast, which can be nephrotoxic in patients with chronic kidney disease [19].

MRI includes imaging pulse sequences that yield diverse tissue contrasts (e.g., T1, T2, water diffusion, steady-state free precession (SSFP), blood flow, and others) without requiring injection of a contrast agent. Although many centers routinely utilize gadolinium-based contrast agents for ADPKD evaluation of complex, hemorrhagic cysts, we have found that this complicates interpretation, prolongs the MRI examination, and doubles the MRI cost if used on every exam. Furthermore, patient anxiety is increased by the use of a gadolinium contrast agent because of their awareness of the nephrotoxicity of CT contrast and warnings regarding nephrogenic systemic fibrosis, even though newer macrocyclic gadolinium agents pose negligible health risks [20].

### 2.2. MRI Protocol Optimization

#### 2.2.1. Magnetic Field Strength, 3 T or 1.5 T

Although 3 T MRI has a high signal-to-noise ratio (SNR), its suitability is diminished in ADPKD patients with massive liver cysts and ascites because dielectric effects at 3 T degrade the image quality. Accordingly, 1.5 T may be preferable when thoracic, abdominal, or pelvic fluid collection is present. However, 3 T head coil coverage for brain magnetic resonance angiography (MRA) is superior when evaluating patients with suspected intracerebral aneurysms [21]. Thus, an imaging center may need two scanners: 3 T for brain MRA and 1.5 T for the remainder of the body imaging.

#### 2.2.2. Extensive Image Coverage with Consistent Breath Holds

To accurately estimate TKV, MR abdominal scans ideally should be acquired with breath holding to prevent respiratory motion artifacts. MR scans for ADPKD often require a large field of view to include the dilated aortic root and arch, enlarged kidneys/liver, and pelvic complications (e.g., free pelvic fluid, seminal megavesicles, and prostate cysts). MRI systems with extensive coil coverage, facilitating a high SNR from the mid-chest into the pelvis, are preferred. This comprehensive coverage, at a reasonable imaging slice thickness, may require multiple breath holds per acquisition. However, when multiple breath holds are utilized, slice-to-slice misalignment of the anatomy (also known as slice misregistration) from inconsistent breath-hold positions can inaccurately represent organ size. To ensure accurate organ volume measurements, MRI technicians should be instructed to prevent excessive overlap between acquisitions and to guide patients to maintain consistent breath-hold positions for each scan [22].

#### 2.2.3. Scanning with Multiple Sequences and Planes

MRI routinely acquires multiple sequences covering organs with various tissue contrasts and in multiple planes (axial, coronal, sagittal). Scanning in multiple planes can overcome inherent partial volume blind spots of each imaging plane (e.g., anterior and posterior blind spots for coronal imaging and superior and inferior for axial imaging). Sagittal acquisitions can boost the spatial resolution by allowing the use of one breath hold for each kidney without worrying about misregistration between breath holds when measuring individual kidney volumes. Routine multi-sequence MR exams for ADPKD can also enhance the organ volume measurement accuracy and reproducibility since averaging organ volume measurements from every MRI sequence mitigates potential biases among the types of imaging tissue contrasts [23]. MRI using multiple imaging sequences also enables the evaluation of dynamic changes in organ imaging features during an MRI exam, which can reflect physiological characteristics (e.g., urinary bladder volume change for urine release, gallbladder volume change for bile production).

An axial 3D spoiled gradient-echo interpolated T1-weighted MRI with a two-point Dixon method produces four sets of images: water only, fat only, in-phase, and out-of-phase, with all slices acquired simultaneously, usually in a single breath hold, covering the abdomen and pelvis. This eliminates slice misalignment issues that degrade T2 fast spin echo and diffusion-weighted imaging (DWI) acquired with the echoplanar technique. Fat quantification is possible from the fat, in-phase, and out-of-phase images. This is important for evaluating hepatic steatosis, lean body mass, and body mass index (BMI) as organ weight increases due to cysts, whereas calorie intake may decrease due to early satiety arising from gastric compression by the enlarged kidneys and liver.

#### 2.2.4. Quality Assurance for Volumetric Analysis

In one study on image-derived biomarkers for ADPKD, about 10% of patients were excluded because of poor imaging quality or artifacts interfering with biomarker quantification [24]. As another quality control measure, a 500 mL saline bag can be placed on the anterior abdomen within the imaging field of view to serve as a volume reference for validating the image-derived volume measurements. Furthermore, if each pulse sequence can be analyzed for organ volumes immediately after acquisition, then outlier volumes can prompt a repeat acquisition. For example, a low organ volume in one sequence compared to other sequences raises the possibility that the organ is not entirely within the field of view. This quality check can allow outlier data to be eliminated or can flag them as needing to be reviewed by an expert observer for a more detailed assessment of whether the data must be discarded.

### 2.3. Post-Acquisition Image Handling

Since a DICOM series may contain DICOM images with different resolutions or incorrect ordering, conversion is required from DICOM to other file formats with a determined resolution and slice ordering, such as NIfTI, for downstream segmentation and radiological reporting. The chosen conversion algorithm and image visualizing software should be robust and capable of managing potential variations in image orientation and voxel order across different pulse sequences and scanner vendors (e.g., dcm2niix [25]). Multi-echo sequences should be broken up into individual images for each echo so that the deep learning algorithm can handle them like other 3D single-echo sequences (e.g., T1, T2). Similarly, DWI images must be divided into a separate series for each b-value. Conversely, if multi-breath-hold acquisition is adopted, “upper” and “lower” acquisition should be combined into one image series. During the combination, preprocessing should be performed so that the volume measurement will be correct. If the series overlap, then the extra overlapping image slices need to be discarded, and if the series have different resolutions, then both series should be resliced to the same resolution.

## 3. Segmentation

### Segmentation Data Collection and Curation

Segmentation refers to labeling image voxels as belonging to an organ or tissue. Manually segmenting (or labeling) organs and cysts is tedious, time-consuming, and a significant barrier to the clinical implementation of deep learning automation of image analysis. A bootstrapped method can help create a large enough labeled dataset. Start by manually labeling a small number of cases from scratch. Use these labeled cases for an initial round of training of a preliminary deep learning model, which can then be used to generate a first iteration of labels on more patients automatically. These labels can then be manually corrected faster than if labeled from scratch. This procedure is repeated to expand our labeled (segmented) dataset for further training and improvement of the deep learning model until the accuracy of the automatic segmentations is acceptable. Manual corrections become easier and less time-consuming in later iterations as the model segmentations become more accurate. This process is known as model-assisted segmentation, and it contributes to iterative model improvements as more training data accumulate.

In our work, we initially only manually labeled the kidneys. This iterative process was later expanded to provide training data on ADPKD patients for most abdominal organs and tissues [23,26], including the following:

- Organs: Native and transplanted kidneys, liver, spleen, pancreas, stomach, gallbladder, urinary bladder, and seminal megavesicles;
- Vascular structures: Aorta and inferior vena cava (IVC);
- Fluid accumulations: Pleural effusion, free pelvic fluid/ascites, and pericardial effusion;
- Cysts: Exophytic renal cysts, hemorrhagic renal cysts, simple renal cysts, hepatic cysts, pancreatic cysts, prostate cysts, and nerve root cysts;
- Body composition: Visceral fat, subcutaneous fat, paraspinal and abdominal wall muscle, and lumbar vertebrae (L1 to L5), each with a distinct label;
- Quality control: External 500 mL saline bag.

The resulting labeled training data are heterogeneous, with variability in the number of scans and modalities for each labeled organ/tissue. Segmenting these anatomical structures requires a versatile multi-label and multi-modality segmentation model, the details of which are beyond the scope of this paper.

Standardizing the labeling is crucial for effectively managing this diverse dataset. Each region of interest (ROI) is assigned a specific label code and color. Labeling criteria are meticulously defined, including the boundaries of each ROI. In this case, segmentation was performed and refined by expert observers (AP, AS, ASF, CZ, DR, EW, HNH, HD, MA, US, VB, ZH), each with experience in labeling > 100 cases, and checked by a board-certified radiologist (MRP) with 30 years of experience in abdominal imaging.

The quality of the organ labeling, whether during the creation of training data or after correcting model outputs, needs to be as good as possible. Software programs that we used to ensure the best-quality labeling included ITK-SNAP 3.8.0 [27], 3D Slicer 5.0.3 [28], and XNAT 1.8.9.1 [29]. In our experience, a good labeling software or platform has short-cuts to (1) adjust the image window level; (2) adjust label opacity: decrease label opacity to visualize the image hidden underneath the label or increase label opacity to screen for segmentation artifacts due to operator error; (3) visualize the original image and reconstructed images in axial, coronal, and sagittal views to screen for image acquisition artifacts or image conversion issues; and (4) selectively overwrite a specific label to refine the editing process.

## 4. Image Biomarkers’ Calculation and Reporting

Three basic imaging metrics are automatically extracted from image segmentations: volume, dimension, and the detection of features (presence or absence). Each biomarker is calculated from the MRI pulse sequences that best demonstrate that metric. Localizer, calibration sequence, DWI, and apparent diffusion coefficient (ADC) maps are excluded due to their low spatial resolution and artifacts (e.g., gradient warping), which can compromise geometric accuracy and render volume measurements unreliable.

### 4.1. Volume, Dimension, and Presence or Absence

Organ volumes are calculated from segmentation by multiplying the voxel volume by the count of voxels in that organ, with the results reported to the nearest cubic centimeter (cm^3^) or milliliter (mL). Reporting may involve volumes from one sequence or an average of volumes from sequences of high quality.

Organ dimensions are determined by placing a 3D bounding box around the organ segmentation and measuring its height, width, and depth. Alternatively, the maximum dimension in any plane or the maximum in a specific direction (e.g., the craniocaudal dimension of the liver) can be provided. These dimensions give insight into the organ shape and are sometimes more practical than volume for small lesions or incompletely captured ROIs (e.g., pleural effusion). They are also more familiar and intuitive for clinicians and patients.

For certain classification features, the main consideration is whether they are present or absent. For example, pancreatic cyst presence may indicate a greater likelihood of PKD2 mutation [30] and should exclude incidental intraductal papillary mucinous neoplasm [31]. The size, border, number, and location might not be clinically relevant. Additionally, some cysts may be too small to measure accurately, especially when the diameter is less than the thickness of the imaging slice. However, for small lesions, it is crucial to document the image series and image slice where the feature is identified to avoid false positive labeling by the radiologist.

The following sections exemplify clinically relevant imaging metrics, including kidney and liver metrics, as well as exploratory metrics, including corrected BMI, urine output, and gastric confinement, which can be derived based on segmentation.

### 4.2. TKV and Its Annual Growth Rate

According to ADPKD management guidelines [32,33], the initial evaluation should include kidney imaging (CT or MRI) to measure the TKV and identify patients at risk of rapid disease progression who may benefit from treatment with tolvaptan. A fast TKV growth rate (>5% per year) derived from serial TKV measurements also indicates rapid disease progression [34]. Yet, critiques highlight the challenges of utilizing TKV growth as a prognostic tool, citing the potential for measurement discrepancies caused by events such as interim cyst rupture and variances in imaging acquisition and measurement techniques [32]. Conversely, other guidelines mention the potential benefit of serial imaging as a crucial tool in monitoring the response to tolvaptan treatment [33].

**Figure 2:**
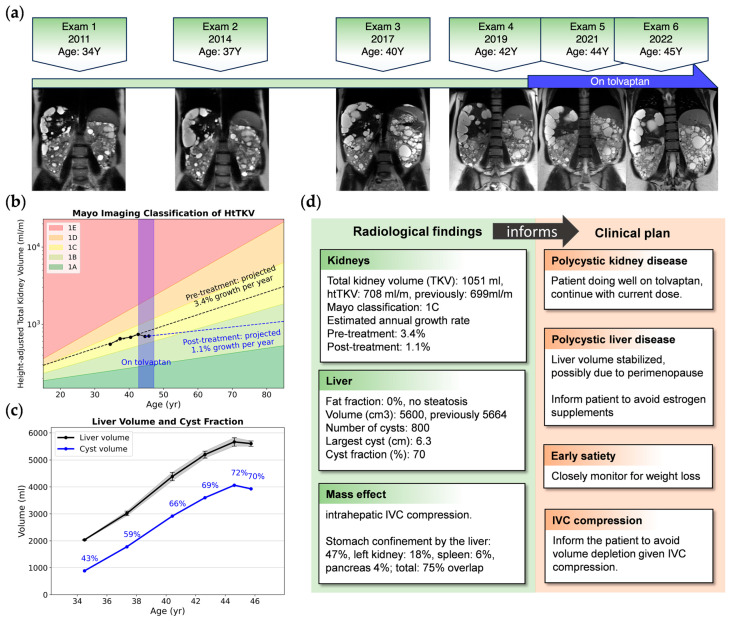
An example of image metrics extracted from longitudinal images and clinical insights for a 34-year-old female with ADPKD, a Mayo class 1C subject who began tolvaptan therapy due to progressive TKV growth. (**a**) Timeline of MRIs showing representative coronal T2 images; (**b**) semi-log plot of htTKV versus age showing TKV growing as predicted by the Mayo Imaging Classification, but the trajectory of extrapolated growth pre-treatment (black dashed line) has a steeper slope compared to post tolvaptan treatment (blue dashed line); (**c**) longitudinal liver volume (black) and total cyst volume with cyst fraction (blue), where liver cyst growth is plateauing, probably due to perimenopause [39]; (**d**) excerpt of a radiological report providing one piece of evidence to guide clinical decision-making. IVC: inferior vena cava; TKV: total kidney volume; htTKV: height-adjusted total kidney volume.

In our practice, we report the TKV over time and the estimated annual TKV growth rate. This growth rate is calculated from serial imaging data [35], presuming exponential kidney enlargement, with intervals of no less than one year between images. Incorporating the growth rate with Mayo Imaging Classification [36] offers a more comprehensive depiction of disease progression, accommodating the inter-individual variability observed within each Mayo class [37]. Moreover, graphical representations of TKV growth trajectories serve as visual aids in evaluating the patient response to treatment (Figure 2).

**Figure 3:**
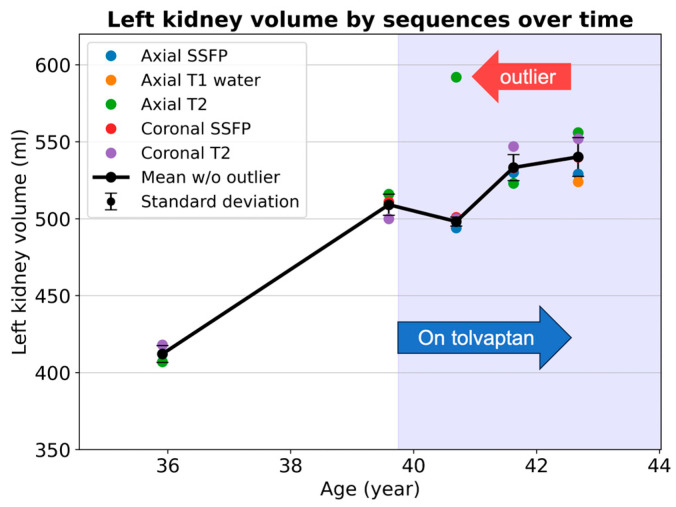
Kidney volume measurements from ages 36 to 43 in an ADPKD patient show good measurement consistency among multiple MRI pulse sequences, except for at age 41, when an outlier value (red arrow) causes a large measurement standard deviation. Standard deviation is illustrated as error bars showing the degrees of measurement uncertainty. The axial T2 images and segmentation should be reviewed to screen for image artifacts or labeling mistakes. Note that the rate of kidney growth decreases following the initiation of tolvaptan in this patient.

When calculating the TKV growth rate, instead of extracting reported volumes from past reports, we extract all sequential MRIs and employ a consistent methodological framework for volume measurements to enhance the reliability [38]: From the earliest available scan to the most recent one, the TKV is segmented via the same model, reviewed by the same observer, using consistent editing software, and reported with a standardized script. This rigorous approach increases the likelihood that follow-up TKV evaluation will remain free from methodological disparities and minimizes observer bias. TKV measurement reproducibility is also enhanced by averaging TKV measurements across multiple sequences within each imaging exam [23]. When plotting the TKV versus time, it is helpful to display all the individual TKV measurements from different sequences at each time point, together with the mean value and standard deviation, to ensure the reliability of each TKV measurement. Variations in the TKV measurements among sequences can trigger the reinterpretation of images and segmentations, to seek out artifacts (Figure 3).

### 4.3. Liver Volume and Liver Cyst Fraction

Polycystic liver disease (PLD) is the most prevalent extrarenal manifestation of ADPKD. Liver cysts are present in 90% of patients with ADPKD aged over 35 [40]. Although PLD rarely causes liver function abnormalities, abdominal discomfort, and other symptoms due to marked enlargement of the liver appear to be prevalent, affecting about 20% of ADPKD patients [32,41]. Additionally, infected liver cysts can be life-threatening. Large, symptomatic cysts offer the opportunity for volume reduction with single-cyst aspiration and sclerosis or fenestration. A partial hepatectomy and liver transplantation are options when there is no dominant cyst suitable for aspiration, but the enlarged liver impairs the quality of life. Thus, a comprehensive radiological report that includes the size and location of the largest cyst, spatial cyst distribution, complex cyst presence, and an assessment of the liver parenchymal reserve becomes instrumental in guiding therapeutic decisions [11,42,43].

Currently, prognostication for PLD is tentative, primarily based on a rudimentary classification including cyst location, size, height-adjusted liver volume, and extent of liver involvement [39,41]. Liver imaging to determine the extent of polycystic liver disease is recommended as a part of the initial assessment of all ADPKD patients [32]. Female ADPKD patients warrant particular attention due to the association of larger liver cysts with the female sex, estrogen exposure, and multiparity [40]. Given the absence of a validated prognostic model and the variable progression of liver disease, particularly in women, regular monitoring of the liver volume and cyst fraction can be helpful, facilitating individualized patient management and timely consideration of invasive (cyst aspiration/fenestration, partial hepatectomy, transplantation) or pharmacological interventions (e.g., somatostatin analogs) [44].

### 4.4. Liver Fat Fraction

Identifying hepatic steatosis is an essential aspect of the ADPKD abdominal MRI exam when patients are being considered for treatment with tolvaptan. Due to tovalptan’s potential hepatotoxicity, liver function tests are required before and after drug initiation [45] since alcoholic and non-alcoholic steatosis are relatively common and may confound the assessment of abnormal liver function tests during tolvaptan treatment. Thus, imaging-based liver fat fraction measurement offers an auxiliary assessment of liver health before initiating tolvaptan treatment. After tolvaptan therapy is begun, measurement of the fat fraction can elucidate the etiology of subsequent increases in liver function tests.

**Figure 4:**
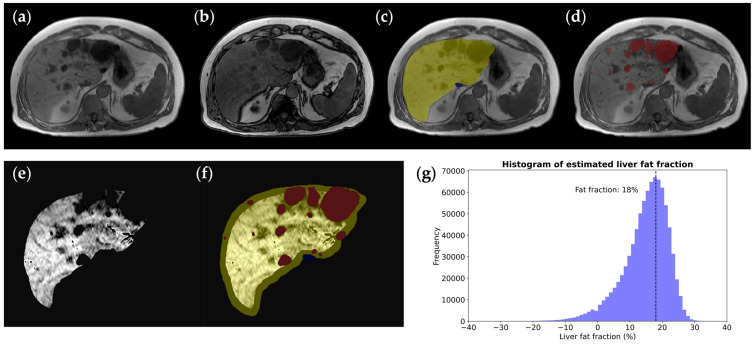
Liver fat fraction estimation in a 62-year-old male ADPKD patient with a (**a**) normal in-phase T1 image but decreased liver signal on (**b**) the out-of-phase T1 image corresponding to fatty infiltration. To calculate the hepatic fat fraction, (**c**) the liver mask (yellow) is taken as the starting point and (**d**) the hepatic cyst mask (red) is subtracted to yield (**e**) a pure parenchymal mask used for measuring the whole liver signal drop-out on out-of-phase images, where the $fat\;fraction=\frac{\left(\text{lliver in-phase signal intensity}\;-\;\text{liver out-of-phase signal intensity}\right)}{2\;\times \;\text{iver in-phase signal intensity}}$. (**f**) A parenchymal mask excluding liver and hepatic cyst segmentation, with erosion at the boundary to exclude peripheral fat tissue. (**g**) A liver voxel signal intensity histogram shows the histogram peak, revealing that there is an 18% calculated liver fat fraction.

Including axial spoiled gradient-echo interpolated T1-weighted images in our protocol with the two-point Dixon method facilitates the quantification of the liver fat fraction. The two-point Dixon method showed no significant difference in liver fat fraction quantification compared to absolute quantification using multi-echo Dixon imaging [46]. Following liver, liver cyst, and IVC segmentation, we derive a refined liver parenchymal mask—excluding liver cysts and major venous structures. By comparing the intensities of the liver parenchymal mask within in-phase and out-of-phase images, the liver fat fraction can be estimated (Figure 4).

### 4.5. Body Composition Quantification

**Figure 5:**
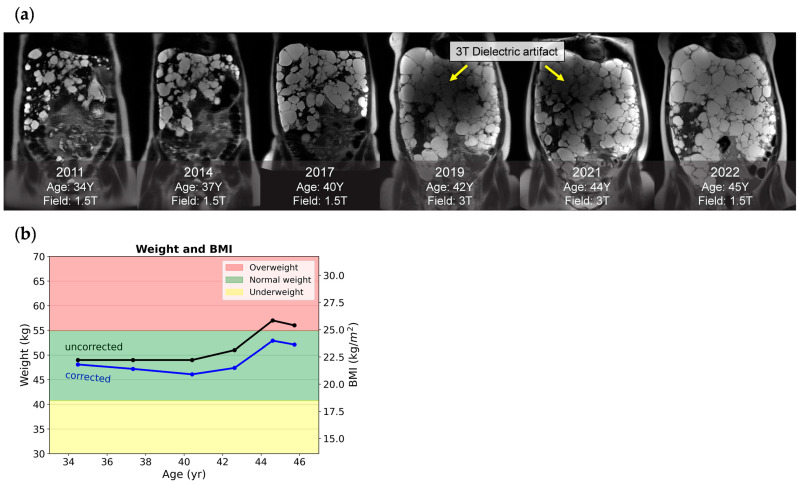
Corrected weight and BMI of a female ADPKD patient with a severe polycystic liver. (**a**) Snapshots of six serial coronal T2 images acquired across 11 years. Notably, images acquired at 3 T in 2019 and 2021 had dielectric artifacts spoiling the image quality, while images acquired from a 1.5 T field with comparable liver cysts in 2022 were free of dielectric artifacts. (**b**) A plot of longitudinal weight and BMI calculated with correction (blue line) and without correction (black line) shows that, from ages 35 to 40, her weight loss was masked by cyst growth. After 44 years, her uncorrected BMI falsely indicated that she was borderline overweight.

BMI-determined obesity (calculated with or without kidney volume) is associated with kidney growth [47] and greater back and radicular pain [48]. However, ADPKD patients may have their BMI overestimated due to a large volume of inert fluid-filled cysts. The patient’s weight and abdominal girth are often increased by these cysts, giving the false impression of being overweight or obese. ADPKD patients may even be advised to lose weight based on their spurious BMI. After segmenting renal and hepatic cysts, the BMI can be corrected by subtracting the estimated cyst weight from the total weight before BMI calculation, assuming a cyst density of 1 g/mL. Figure 5 shows an example of a female with progressive PLD. Images spanning 11 years show the enlarging liver cysts masking weight loss from the ages of 34 to 40. On her most recent exam, 4 L of kidney/liver cysts accounted for approximately 4 kg of extra weight. Her BMI without correction (25.8 kg/m^2^) falsely indicated that she was borderline overweight, but after correction by excluding kidney/liver cysts’ weight, her weight was in the normal range.

The BMI is not necessarily associated with adiposity, even in the general population [49]. Thus, some studies use imaging to quantify adipose and muscle mass, to identify the subtle differences that the BMI may otherwise mask. One study found that abdominal visceral adiposity, quantified by coronal MRI, was independently associated with faster kidney growth and improved kidney growth prediction in individuals with a normal BMI [50]. Another study quantified abdominal muscle mass at the third lumbar spine vertebra level using MRI or CT and found that ADPKD patients had a higher rate of sarcopenia. At the same time, a previous study found no significant differences in BMI categories between ADPKD patients and controls [51].

### 4.6. Urine Output and Ureteral Jet Effect

The urine output is a critical indicator of renal function, with the typical range between 0.5 and 1.5 mL/kg/hour [52]. For patients with ADPKD, this measure can vary significantly due to factors affecting kidney function [53] and treatment effects [54,55]. Clinical trials have noted marked increases in the 24 h urine volume to a median of 6 L/day during tolvaptan treatment [54,55,56]. Furthermore, increased hydration—recommended at approximately 2 to 3 L daily—can suppress vasopressin levels and potentially slow ADPKD progression [57]. Thus, the urine output is a potential metric for checking the patient’s adherence to hydration recommendations.

**Figure 6:**
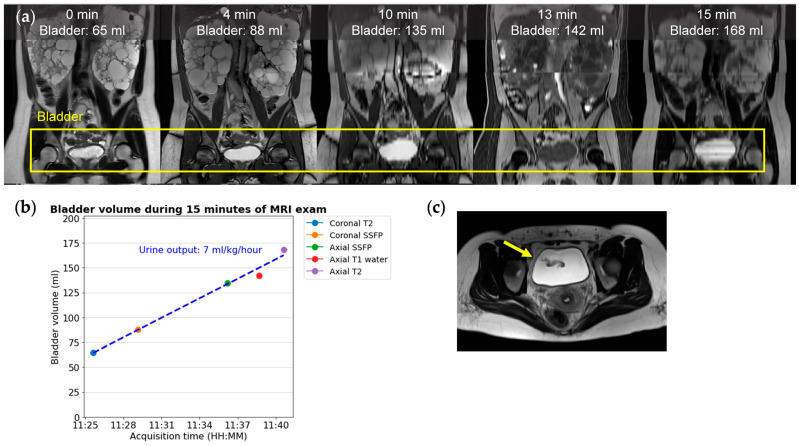
In this 36-year-old ADPKD female patient, the urine output is 7 mL/kg/hour, estimated from serially acquired images during a 15 min MRI exam of 5 sequences; (**a**) from left to right: coronal T2, coronal SSFP, coronal reformation of axial SSFP, T1, and T2. (**b**) A graph of urine output corresponding to the slope of a linear regression fit to the bladder volume divided by the subject’s weight. The DICOM image timestamp is treated as the instantaneous acquisition time for each image. In our experience, T1 (red dot) commonly shows a lower bladder volume compared to other sequences. (**c**) An axial T2 image with a ureteral jet in-flow signal void artifact (yellow arrow).

The bladder volume increasing throughout the imaging exam corresponds to urine output (Figure 6). Imaging-based urine output estimation necessitates precise organ segmentation across various sequences alongside established data collection and analysis protocols. Considerations include the sequence order, the impact of diurnal variations, such as a higher observed urine output in the evening [54], and the dose and half-life of tolvaptan [58], which complicate spot measurements compared to 24 h totals [59]. Despite these challenges, leveraging routine MRI exams for urine output estimation presents an opportunity to enrich ADPKD management with critical, non-invasive measurements.

Once the bladder is segmented, it typically has a homogeneous T2 bright signal typical of water. However, where pulses of urine enter the bladder from each ureteral orifice, the fast-flowing ureter jets create signal void artifacts (Figure 6c). When patients are well hydrated and have a greater urine output, larger and more frequent ureteral jet artifacts are expected. Identifying these artifacts as an increase in signal intensity standard deviation or through labeling has the potential to enable the routine identification of asymmetry of renal function.

### 4.7. Gastric Confinement

**Figure 7:**
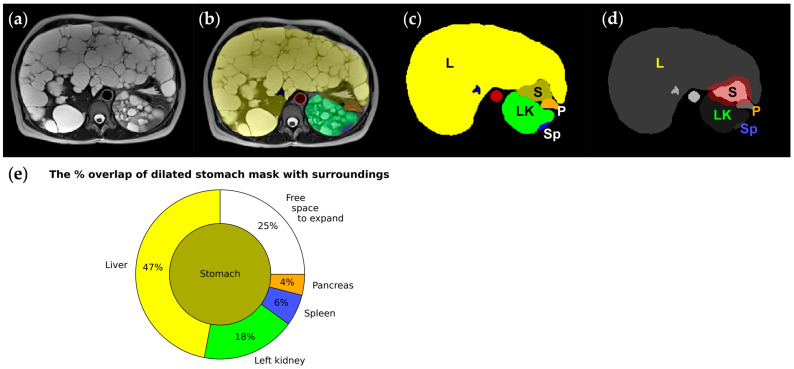
Images and segmentation from a 45-year-old female ADPKD patient with early satiety (**a**–**c**), where the liver (L: yellow) and left kidney (LK: green) enlarged by hundreds of cysts squeeze the stomach (S: greenish brown); (**d**) stomach mask dilated circumferentially by 10 mm (red) to simulate postprandial distension, demonstrating how surrounding organs must be pushed out of the way for the stomach to expand. (**e**) A pie chart graphically showing the amount of the stomach’s adjacent space occupied by the liver, left kidney, spleen, and pancreas that needs to be pushed out of the way for stomach expansion. Only 25% of the stomach does not touch adjacent organs and is free to expand.

Early satiety is one of the most prominent symptoms of polycystic liver disease. MR-based morphological analysis of the stomach during gastric filling and emptying has found that expansion of the proximal stomach is constrained posteriorly by the pancreas and spinal column and laterally by the liver and spleen. In contrast, anterior and inferior expansion is relatively unhindered [60]. Thus, from the imaging and segmentation perspective, the extent of the gastric spatial restriction can be quantified by artificially expanding the stomach segmentation by approximately 10 mm on axial images to simulate postprandial distension. Subsequently, the percentage of volume overlap of this expanded stomach segmentation with the segmentation of adjacent organs, such as the liver, left kidney, pancreas, and spleen, is measured (Figure 7). Future studies are warranted to determine the appropriate simulation approach to represent compartmentalized and asymmetric characteristics of stomach postprandial expansion and torsion [60,61], evaluate the reproducibility of stomach confinement measurements, and establish the correlation between this imaging-based metric and the subjective experience of early satiety. Additionally, it may be beneficial to investigate the prognostic utility of this metric in predicting the onset of early satiety among ADPKD patients.

## 5. Summary

“Images are data” [62]. Medical images are vast sources of information that are rarely used to their full potential. Now that AI and deep learning model segmentation of kidneys are beginning to be used to extract the critical biomarkers of the total kidney volume, it is exciting to explore the possibilities for automatically extracting additional information from MRI exams by building upon the deep learning model segmentation approach. Organ segmentation reveals the structures of all organs and tissues and additional features that reflect their function. Since segmentation is automatic, it is easy to apply to all prior exams, to track how these metrics change over time, without worrying about methodologic confounders. These automated metrics calculated from organ and tissue segmentation complement the rich information from MRI studies already produced through radiologist interpretation [13], including the detection of renal masses, cyst rupture, infected cysts, anatomical variations, pancreatic cysts, seminal megavesicles, umbilical, hiatal, inguinal, and other hernias, tumors involving the liver and other organs, hepatic biliary ductal dilatation, IVC compression, degenerative disk disease, dural ectasia, and findings that can explain patient symptoms including sources of pain.

Other studies have reported ambitious attempts to segment everything in medical imaging. Notably, “TotalSegmentator”, released in 2022, provides a semi-robust segmentation of 104 anatomical structures in CT images [63]. Additionally, an MRI-based abdominal organ segmentation model was applied across a cohort of 20,000 subjects, suggesting the capability of the segmentation model to handle large datasets [64]. However, these prior studies failed to take the next step of using segmentation to calculate more clinically meaningful metrics, as illustrated here.

Future directions may involve segmenting more organs/tissues to calculate imaging metrics. In the quest for the best prognostic imaging biomarker in ADPKD, one impressive study segmented each renal cyst to quantify the total cyst number and cyst parenchymal surface area, an approach that outperformed the TKV in predicting the slope of estimated glomerular filtration rate decline [24]. Other potential metrics of interest include the skeletal muscle index, which is useful for sarcopenia screening [51] and for correcting estimated glomerular filtration rates [65]; the fat index, for characterizing abdominal adiposity [47]; seminal vesicle lumen measurements, enabling the identification of seminal megavesicles, the automated detection of an aortic aneurysm, and the assessment of aorta pulsatility; and metrics for cardiac size and left ventricular mass, which could contribute to screening and monitoring cardiovascular complications of ADPKD.

For these exploratory metrics, it is crucial to enforce image quality control measures to identify any image artifacts that could disrupt the metrics’ measurement. Such artifacts include breathing motions and slice misregistration, which may affect volumetry, as well as intensity-based artifacts like partial fat–water swaps, which can influence the quantification of the organ fat fraction. Assessing the reproducibility of these metrics through test–retest evaluation is important, considering variations in acquisition time, imaging parameters, and short-term factors such as food or fluid intake [66]. Gastric confinement evaluation should encompass diverse stomach volumes, filling statuses, imaging planes, and resolutions, while uncertainty in urine output linear regression estimation should be assessed across different starting bladder volumes and acquisition durations. Comparative analyses against established standards are also necessary; for instance, liver fat fraction quantification should be validated against magnetic resonance spectroscopy, and the dimensions of the aorta and left ventricle should be compared with echocardiography-derived values, CT images, or ECG-gated cardiac MRI. Additionally, the clinical significance and prognostic utility of these metrics warrant investigation: for instance, assessing whether the image-based urine output in uncontrolled conditions offers comparative values to a spot urine osmolarity check and reflects the hydration status, and evaluating whether gastric confinement accurately reflects early satiety levels or predicts future instances of early satiety. Despite the need for a comprehensive evaluation, we propose that these imaging metrics hold promise and that their use will provide tangible benefits to patients.

## Data Availability

Anonymized data is available upon request under a data usage agreement.

## Abbreviations

ADC	Apparent diffusion coefficient
ADPKD	Autosomal dominant polycystic kidney disease
AI	Artificial intelligence
BMI	Body mass index
CRISP	Consortium for Radiologic Imaging Studies of Polycystic Kidney Disease
CT	Computed tomography
DWI	Diffusion-weighted imaging
FDA	U.S. Food and Drug Administration
htTKV	Height-adjusted total kidney volume
IVC	Inferior vena cava
MIC	Mayo Imaging Classification
MRA	Magnetic resonance angiography
MRI	Magnetic resonance imaging
NIfTI	Neuroimaging Informatics Technology Initiative
PLD	Polycystic liver disease
ROI	Region of interest
SNR	Signal-to-noise ratio
SSFP	Steady-state free precession
SSFSE	Single-shot fast spin echo
TKV	Total kidney volume
